# Evaluation of cytotoxicity of bevacizumab on VEGF-enriched corneal endothelial cells

**Published:** 2011-12-20

**Authors:** Raluca Rusovici, Monali Sakhalkar, Kakarla V. Chalam

**Affiliations:** Department of Ophthalmology, University of Florida, College of Medicine, Jacksonville, FL

## Abstract

**Purpose:**

To evaluate the cytotoxicity of varying doses of Bevacizumab on corneal endothelial cells in the presence of a range of concentrations of vascular endothelial growth factor (VEGF). Bevacizumab, a drug widely used in the treatment of neovascular glaucoma neutralizes all isoforms of VEGF and ameliorates neovascularization after intracameral administration. However, the safety of intracameral administration of Bevacizumab and dose–dependent toxicity on corneal endothelial cells has not been established.

**Methods:**

Bovine corneal endothelial (BCE) cells were treated with VEGF (50 ng/ml) and/or Bevacizumab (0.1–2 mg/ml) for 72 h. Cell proliferation was measured with the water soluble tetrazolium salts (WST-1) assay. Morphological changes were recorded by bright-field microscopy of cells. Cytotoxicity in response to Bevacizumab was evaluated by trypan blue exclusion, as well as annexin V/propidium iodide (PI) staining.

**Results:**

Bevacizumab was not cytotoxic at the concentrations tested and the percentage of Bevacizumab-treated cells staining positively for both PI and Annexin V was less than 1%. The anti-proliferative effects of Bevacizumab on BCE cells were dose-dependent; a dose of 1.5 mg/ml or 2 mg/ml produced a 33% (p=0.005) or 47% (p=0.001) decrease in cell proliferation compared to controls. Similar results were obtained in cells treated with a combination of Bevacizumab and VEGF. VEGF (50 ng/ml) had no significant effect on cell proliferation compared to controls. Morphology of cells was unchanged after treatment with Bevacizumab and/or VEGF compared to controls.

**Conclusions:**

Bevacizumab was safe and not toxic to BCE cells at concentrations commonly used in clinical practice.

## Introduction

Bevacizumab, a full-length, humanized, anti- vascular endothelial growth factor (VEGF) monoclonal antibody has shown promising success in the treatment of age-related macular degeneration, choroidal neovascularization and proliferative diabetic retinopathy [1-3]. Topical Bevacizumab is used in early bleb failure after trabeculectomy, corneal neovascularization after penetrating keratoplasty and progressive corneal neovascularization resistant to conventional therapy [4-6]. In addition, Bevacizumab effectively inhibits iris neovascularization in neovascular glaucoma after intracameral administration [7,8].

However, the safety of intracameral administration of Bevacizumab and dose-dependent toxicity on corneal endothelial cells have not been established. Toxicity to the corneal endothelial cells can lead to loss of corneal transparency and consequential blindness.

We evaluated the cytotoxicity of varying doses of Bevacizumab on corneal endothelial cells independently, as well as in association with VEGF in vitro. Varying concentrations of VEGF were used to mimic aqueous dynamics of neovascular glaucoma.

## Methods

### Cell culture

Bovine corneal endothelial (BCE) cells were purchased from ATCC (Manassas, VA) and plated according to the manufacturer’s protocol. The stock cell cultures were maintained in T-75 flasks in Dulbecco Minimum Essential Medium (DMEM; Invitrogen, Carlsbad, CA), supplemented with 10% fetal bovine serum (FBS; Sigma-Aldrich, St. Louis, MO) containing 100 mg/ml penicillin and 100 mg/ml of streptomycin (Invitrogen) at 37 °C in a 95% air and 5% CO_2_ incubator. BCE cells were treated with different concentrations of human vascular endothelial growth factor (0–100 ng/ml; VEGF_165_; Pepro Tech, Rocky Hill, NJ) and/or 0.1–2 mg/ml Bevacizumab (Avastin®; Genentech, South San Francisco, CA), a recombinant humanized monoclonal antibody that inhibits the biologic activity of human VEGF (Pepro Tech), for 72 h.

### Cell cytotoxicity

#### Trypan blue exclusion assay

Cytotoxicity was evaluated by trypan blue exclusion assays. To test whether our treatment with Bevacizumab at the doses and time points measured was cytotoxic, we performed trypan blue staining using an automated cell counter. Parallel experiments with cell proliferation assays were set up in 6-well dishes by plating 10,000 cells/well. Cells were allowed initially to attach for 24 h. The cells were treated similarly as cells for proliferation studies, with Bevacizumab alone (0.1, 0.5, 1.0, 1.5, 2 mg/ml) or in combination with VEGF (50 ng/ml). After treatment, cells were trypsined and centrifuged at 1,400× g for 5 min. The cell pellets were resuspended in 0.5 ml DMEM and counted. Counting was performed using the ViCell XR Cell Viability analyzer (Beckman Coulter, Fullerton, CA) according to the manufacturer’s protocol.

#### Morphology

Before exposure of corneal endothelial cells to Bevacizumab, cellular morphology was recorded by bright-field microscopy. Subsequently, cell morphology was assessed with an Olympus IX51 microscope (Olympus, Centre Valley, PA), 72 h after incubation with respective concentrations of Bevacizumab (0.1, 0.5, 1, 1.5, 2 mg/ml), VEGF (50 ng/ml) plus Bevacizumab and VEGF alone. Signs of gross cellular damage, such as changes in cytoplasmic and nuclear morphology as a result of cytotoxicity were assessed, in both control and treated cells [9]. Serum-starved cells served as controls.

#### Flow cytometry

Corneal endothelial cell cytotoxicity was assessed by flow cytometric evaluation of annexin V fluorescein isothiocyanate (FITC)-conjugate, propidium iodide (PI)- staining cells. For flow cytometry assays 100,000 cells/well were plated in 6-well plates. The cells were allowed to attach for 24 h. The cells were treated with varying concentrations of Bevacizumab (0.1, 0.5, 1.0, 1.5, 2 mg/ml) and/or VEGF (50 ng/ml) and incubated for another 72 h. For flow cytometry assays using PI only, the cells were trypsinized and the pellet was resuspended in 70% ethanol (Sigma-Aldrich) solution for 10 min at 4 °C. After centrifugation and subsequent washes with Hank’s Balanced Salt Solution (HBSS) (Invitrogen), the pellet was resuspended in 10 µg/ml PI (Sigma-Aldrich) solution and incubated at room temperature for 30 min in the dark [10]. Annexin V labeling was used to detect apoptotic cells (Sigma-Aldrich). After centrifugation and subsequent washes with Hank’s Balanced Salt Solution (HBSS; Invitrogen), the pellet was resuspended in 1× Binding buffer (Sigma-Aldrich). Cells incubated with annexin V-FITC and PI solution for 10 min at room temperature were immediately analyzed. Cells, which were early in the apoptotic process, stained with annexin V-FITC conjugate alone. By this method, necrotic (dead cells) stain with both PI and annexin V-FITC. Stained cells were analyzed on a Beckman Coulter (Fullerton, CA) flow cytometer using excitation at 488 nm and emission at 600 nm for PI and 494 nm and 520 nm for annexin-FITC, respectively. Ten thousand cells were counted per sample, and the data was processed using standard software (Beckman Coulter).

#### Cell growth assays

For cell growth assays, cells were plated at a density of 3,000 cells/well in 96-well plates. Cellular proliferation was assessed according to the manufacturer’s instruction with the 4-[3-(4-lodophenyl)-2-(4-nitrophenyl)-2H-5-tetrazolio]-1.3-benzene disulfonate (WST-1) kit (Roche, Mannheim, Germany). The colorimetric assay is based on the cleavage of the tetrazolium salt WST-1 by mitochondrial dehydrogenases in viable cells. WST-1 solution (100 µl/well) was added to cells in 96-well plates followed by incubation for 2 h at 37 °C. The plate was read on a spectrophotometer at 440 nm with a reference wavelength at 690 nm.

To determine the safety of Bevacizumab on corneal endothelial cells, we performed WST-1 proliferation assays. Cells were treated with 0–100 ng/ml VEGF and / or different doses of Bevacizumab (0.1, 0.5, 1, 1.5, 2 mg/ml) for 72 h. The concentrations of Bevacizumab used were clinically relevant [11]. VEGF was added to simulate the anterior chamber microenvironment that is often seen in neovascular glaucoma.

### Statistical analysis

All experiments were done in at least triplicates. Statistical analysis among treatment groups was performed with ANOVA (GraphPad, La Jolla, CA). For proliferation and viability assays two-tailed *t*-test analysis was used to determine p-values. For flow cytometry, results were presented as means+/−standard error.

## Results

### Cell cytotoxicity

#### Trypan blue exclusion assay

**Figure 1:**
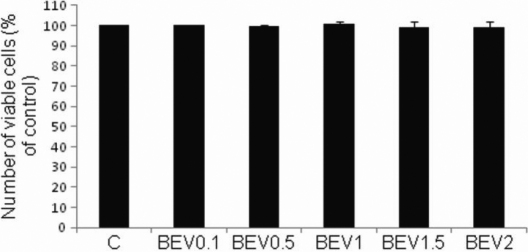
Effect of different concentrations of Bevacizumab on BCE cell viability. Cell viability was determined by trypan blue exclusion assays on an automated cell counter. Cell viability is unchanged between Bevacizumab treated samples and controls. Viability rates are expressed as percentages of control values. BEV (Bevacizumab).

In controls, corneal endothelial cell viability was 98%, in Bevacizumab (0.1, 0.5, 1, 1.5, 2 mg/ml) treated cells viability was 98%, 97.3%, 98.1%, 94.8%, and 94.2%, respectively. The differences in viability were non–significant (p=0.4426, 0.4426, 0.4426, 0.839, 0.785, respectively, compared to controls) and Bevacizumab was not cytotoxic at the concentrations tested (Figure 1). In VEGF (50 ng/ml)-treated cells viability was 98.3% (p=0.6479). In Bevacizumab (0.1, 0.5, 1, 1.5, 2 mg/ml) and VEGF (50 ng/ml) treated samples the viability was 98.3%, 98.3%, 99%, 98%, 97% and 95.4%, respectively. VEGF (100 ng/ml) did not induce cytotoxicity (97% viability compared to 98% viability in controls). VEGF (50 ng/ml) and Bevacizumab treated cells at all concentrations tested (0.1, 0.5, 1, 1.5, 2 mg/ml) showed 95.4%–99% viability compared to 98% viability in control groups (p=0.845, 0.99, 1, 0.40, 0.55, respectively, unpublished data).

#### Morphology

**Figure 2:**
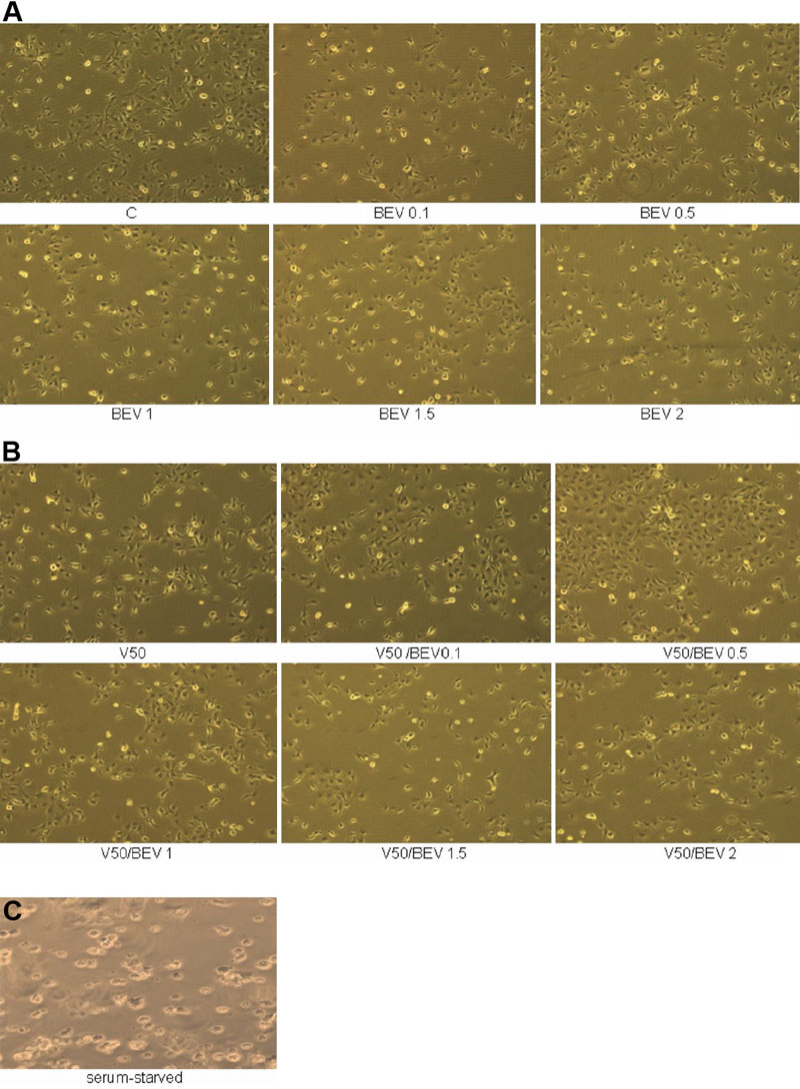
Effect of Bevacizumab and VEGF on BCE cell morphology. **A**: Effect of different concentrations of Bevacizumab alone on BCE cell morphology. **B**: Effect of different concentrations of Bevacizumab and VEGF on BCE cell morphology. Photomicrographs showing no observable differences in morphology among different treatment groups and controls. Bright-field images were taken at a 20× magnification. **C**: Serum-starved BCE cells showing small, picknotic nuclei, shrunken cytosol served as positive controls. BEV (Bevacizumab); V (vascular endothelial growth factor; VEGF).

Bright-field microscopy of polygonal BCE cells in culture after 72 h did not show cell membrane damage, a shrunken cytosol or nuclear changes in either controls, VEGF, Bevacizumab alone and VEGF plus Bevacizumab-treated groups at all concentrations tested (0.1, 0.5, 1, 1.5, 2 mg/ml Bevacizumab; 50 ng/ml VEGF; Figure 2A,B). Serum- starved cells in contrast showed rounded cells with picknotic nuclei and minimal cytosol (Figure 2C).

#### Flow cytometry

**Figure 3:**
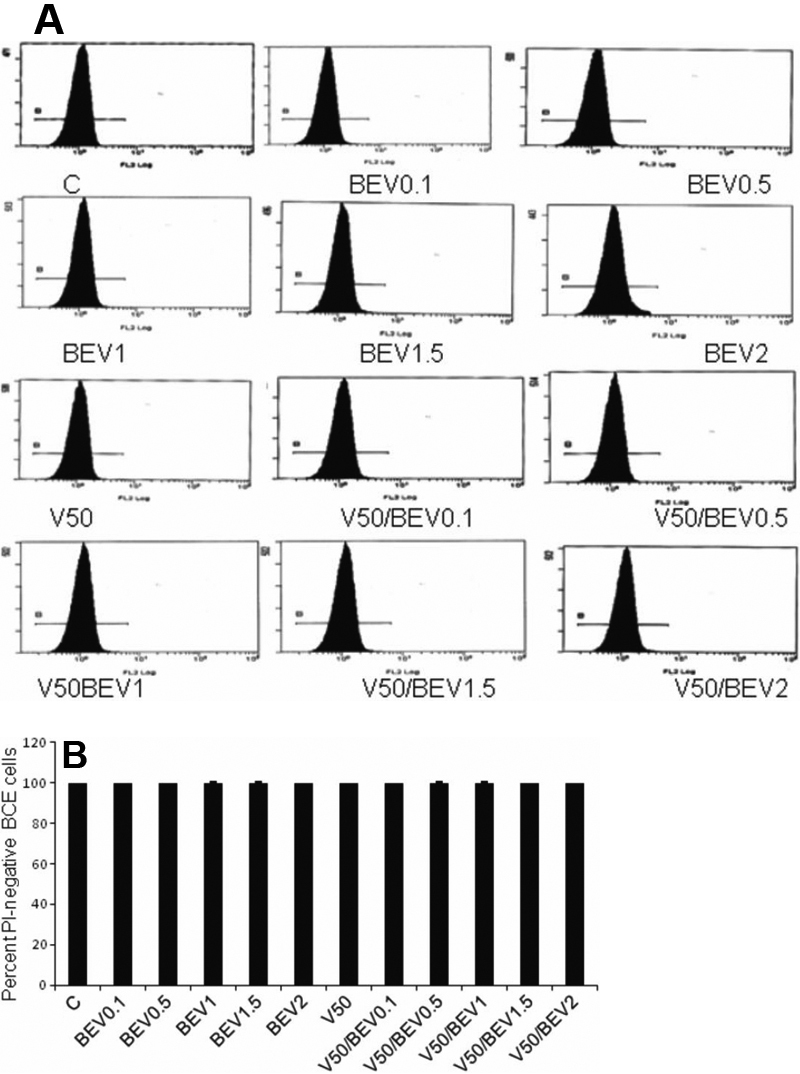
Effect of Bevacizumab on BCE cell death as quantified by flow cytometry. **A**: Flow cytometry analysis of BCE cells treated with Bevacizumab and/or VEGF. Cells were stained with propidium iodide (PI) before flow cytometry analysis. **B**: Viable percentage of gated cells (Non-PI staining) from experiment shown in **A**. BEV (Bevacizumab); V (vascular endothelial growth factor; VEGF).

In flow cytometry experiments where only PI staining was used, cytotoxicity of cells treated with VEGF (50 ng/ml) was similar to controls (p=0.2909; Figure 3A,B). Bevacizumab did not lead to cytotoxicity in BCE cells and PI incorporation in cells treated with Bevacizumab was similar to controls (p=0.2752). The average percentages of cells staining negatively for PI were 99.5% (controls), 99.6% (50 ng/ml VEGF-treated cells), 99.5%, 99.6%, 99.5%, 99.7%, 99.8% (0.1 mg/ml, 0.5 mg/ml, 1 mg/ml, 1.5 mg/ml, 2 mg/ml Bevacizumab-treated cells, respectively) and 99.9%, 99.4%, 99.5%, 99.6%, 99.7% (50 ng/ml VEGF and 0.1 mg/ml, 0.5 mg/ml, 1 mg/ml, 1.5 mg/ml, 2 mg/ml Bevacizumab-treated cells, respectively; Figure 3A,B).

**Figure 4:**
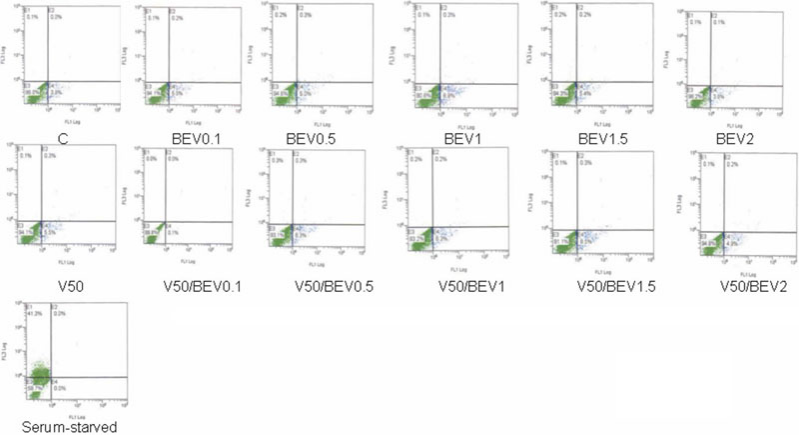
Effect of different concentrations of Bevacizumab on BCE cell death as quantified by flow cytometry. Flow cytometry analysis of BCE cells treated with Bevacizumab and/or VEGF. Cells were stained with annexin V/propidium iodide (PI) before flow cytometry analysis. Percentages of annexin V/PI negative (viable cells) are displayed in the lower left quadrant; annexin V negative/PI positive cells are displayed in the upper left quadrant. Percentages of annexin V positive/PI positive (necrotic) cells are displayed in the upper right quadrant. Percentages of annexin V positive/ PI negative (early apoptotic) are displayed in the lower right quadrant. Cells under serum-starved conditions were considered as positive controls. BEV (Bevacizumab); V (vascular endothelial growth factor; VEGF).

The percentages of cells staining positively for PI and Annexin V, representing the population of cells which had undergone necrosis were less than 1% (0.0%–0.3%) across control (0.0%), VEGF alone (0.3%), Bevacizumab alone (0.2%, 0.3%, 0.3%, 0.2%, 0.1%, respectively) and Bevacizumab and VEGF (0.0%, 0.3%, 0.2%, 0.3%, 0.2%, respectively) treatment groups (Figure 4).

To further determine whether Bevacizumab produced more subtle cytotoxicity in these cells, such as early apoptotic changes, cells staining with annexin V, but not PI were analyzed. The percentages of control and Bevacizumab (0.1, 0.5, 1.0, 1.5, 2 mg/ml)-treated cells undergoing early apoptotic death are comparable (3.9% in controls compared to 5.5%, 5.0%, 8.9%, 5.4%, 3.6% in Bevacizumab-treated cells, respectively). The percentages of VEGF alone and Bevacizumab (0.1, 0.5, 1.0, 1.5, 2 mg/ml) and VEGF-treated cells undergoing early apoptotic death were 5.5% in VEGF-treated cells compared to 0.1%, 6.3%, 6.3%, 8.5%, 4.9% in Bevacizumab-treated cells, respectively (Figure 4). Annexin V, PI-double negative (non-apoptotic) cells accounted for 96% of controls, 94% of VEGF (50 ng/ml)-treated cells and 94.1%, 94.6%, 90.6%, 94.3%, 96.2%, respectively, of Bevacizumab-treated cells. Similarly, percentages of Bevacizumab (0.1, 0.5, 1, 1.5, 2 mg/ml) and VEGF-treated annexin V, PI-double negative cells were 99.8%, 93.1%, 93.2%, 91.1%, 94.8%, respectively. In comparison, cells under serum-starved conditions (positive controls) showed 41.3% AnnexinV/PI -positive staining (Figure 4). These results are similar to those obtained through cell viability analysis studies across control and treatment groups.

#### Cell growth assays

**Figure 5:**
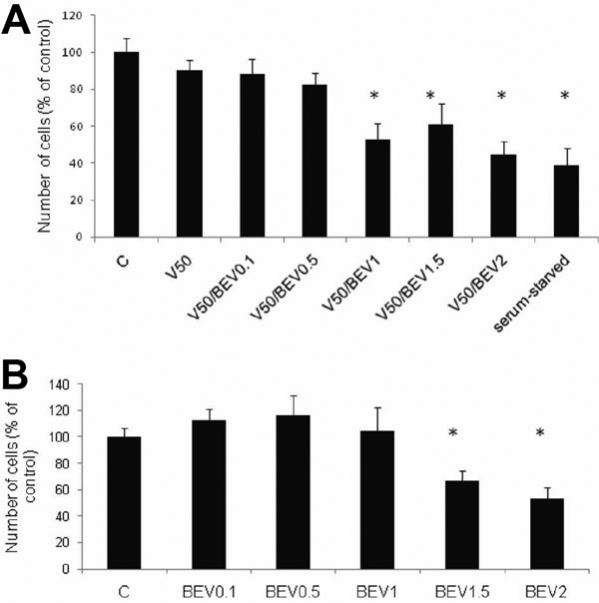
Effect of VEGF and Bevacizumab on proliferation of BCE cells. **A**, **B**: Effect of different concentrations (0.1–2 mg/ml) of Bevacizumab on proliferation of BCE cells. Increasing concentrations of Bevacizumab (1–2 mg/ml) produce a significant decrease in cell proliferation rates compared to controls. VEGF does not significantly change BCE proliferation rates as compared to controls. Cell proliferation was determined by the WST-1 assay and automated cell counting. Proliferation rates are expressed as percentages of control values. Proliferation rates under serum-starved conditions were considered as positive controls; BEV (Bevacizumab); V (vascular endothelial growth factor; VEGF). * Indicates a significant decrease as compared to control (p<0.05).

Proliferation rates of treatment groups were quantified as percentages of control proliferation values (which were considered 100%). Proliferation rates in VEGF-treated samples were 90.4% of those in control samples (VEGF 50 ng/ml; p=0.32; Figure 5A,B). We observed an increase in cell numbers (a 20% increase compared to controls) in response to VEGF at 100 ng/ml (p=0.115; unpublished data).

Proliferation rates in Bevacizumab (0.1 mg/ml, 0.5 mg/ml, 1 mg/ml)-treated cells were 85.5%, 84.7%, 95.7%, respectively, compared to control proliferation rates (p=0.255, 0.318, 0.83, respectively). Bevacizumab (1.5 mg/ml or 2 mg/ml) produced a significant 33% (p=0.005) or 47% (p=0.001) decrease in cell proliferation rates compared to controls. VEGF (50 ng/ml) and Bevacizumab (0.1, 0.5 mg/ml)-treated cells showed decreased proliferation levels (88%, 82% compared to controls, respectively) as did Bevacizumab-only treated cells at the same concentrations (p>0.05; Figure 5A,B). For VEGF and Bevacizumab (1.5, 2 mg/ml)-treated samples there were significant decreased proliferation rates compared to controls (60%, 44% compared to controls, respectively; p=0.0114, 0.001). The decreases in proliferation rates for both Bevacizumab (1.5, 2 mg/ml) and Bevacizumab plus VEGF samples were comparable. Serum-starved cell proliferation rates were 38.71% of control values. Positive controls (serum-starved cells), showed a significant decrease in cell numbers compared to controls (p<0.01).

## Discussion

Anti-VEGF treatment has revolutionized the management of vasoproliferative diseases of the retina, such as choroidal neovascularization associated with age related macular degeneration, proliferative diabetic retinopathy, macular edema associated with central retinal vein occlusion and neovascular glaucoma [12].

The pharmacokinetics of Bevacizumab after an intracameral injection are not well known. Intracameral Bevacizumab may metabolize faster from the anterior chamber than after an intravitreal injection and hence, may be less cytotoxic to the corneal endothelium. However, it is important to evaluate the effect of Bevacizumab on the corneal endothelium, since corneal endothelial cells are likely to be exposed to Bevacizumab after intracameral injection for a considerable time period.

Corneal endothelial cell count declines steadily with age [13]. Endothelial dystrophy, inflammation and trauma can result in more serious cell loss. Lower endothelial cell count leads to loss of corneal transparency and consecutive corneal edema. A rapid increase in the aqueous Bevacizumab concentration is observed when Bevacizumab eye drops were instilled in rabbit corneas, suggesting that Bevacizumab eye drops can sufficiently penetrate the corneal stroma and the anterior chamber [14]. In our study we examined the effect of increasing concentrations of Bevacizumab on bovine corneal endothelial cells in the presence of vascular endothelial growth factor.

In normal human corneas from donor eyes, VEGF is expressed in corneal epithelial and endothelial cells, vascular endothelial cells of the limbal vessels and keratinocytes [15]. VEGF receptors are expressed in increased densities in inflamed and vascularized corneas compared to normal corneas and thus may play an important role in corneal neovascularization [15]. The surrounding aqueous humor provides the cornea with nutrients including VEGF. Intracameral administration of Bevacizumab neutralizes VEGF available to the corneal endothelial cells. Bevacizumab may be delivered topically, intracamerally or intravitreally for treatment of ocular neovascularization [10,16]. Among the different types of delivery, intracameral delivery is used less frequently. Topical treatment with Bevacizumab in rabbit eyes leads to aqueous accumulation of the drug [14]. Intravitreal and intracameral delivery is equally effective in regression of iris vascularization [17,18,]. In a retrospective study of 1,200 patients treated with intravitreal Bevacizumab, corneal infiltrative keratitis and corneal stromal edema were noted in 1.1% of patients [19].

Chalam et al. [11] demonstrated that Bevacizumab was nontoxic to human corneal epithelial, corneal fibroblast and human umbilical vascular endothelial cells at various doses. Bevacizumab was not toxic to human corneal endothelial cells at a concentration of 5 mg/ml [20]. Hosny et al. [21] showed statistically significant high endothelial cell loss (3.4%) after intracameral injection of Bevacizumab in vivo; however the corneal transparency was not affected and remained clear.

Bevacizumab has been employed intracamerally at a 1.5 mg and 2.5 mg dose (0.06 ml or 0.1ml of a 25 mg/ml solution, respectively) [22-24]. We selected our doses of Bevacizumab at 0.1, 0.5, 1, 1.5 and 2 mg/ml to encompass two log units above and two log units below the current clinical range. We used bovine corneal endothelial cells for our experiment which resemble human corneal endothelial cells. These results are consistent with the non-cytotoxicity of Bevacizumab on other cell types [11,25]. In our study, Bevacizumab (1.5 mg/ml or 2 mg/ml) produced, respectively, a 33% (p=0.005) or 47% (p=0.001) decrease in cell proliferation rates compared to controls. The anti-proliferative effects of Bevacizumab in BCE cells were dose-dependent (1–2 mg/ml). Our study showed no cytotoxicity with Bevacizumab (0.1, 0.5, 1, 1.5 and 2 mg/ml).

Cytotoxic compounds alter the morphology of corneal endothelial cells. Pleomorphism and nuclear enlargement of corneal endothelial cells are noted in response to increasing doses of recombinant tissue plasminogen activator [9]. Bevacizumab did not alter the cellular morphology of corneal endothelial cells despite incubation with various concentrations for 72 h. In contrast, serum-deprived BCE cells, which served as positive controls, were rounder and smaller.

Intracameral injections of Bevacizumab in rabbit eyes at concentrations of 1.25 mg per 0.05 ml solution for 1 month were not toxic to corneal endothelium [26]. Our results were similar in a cell culture model.

In conclusion, Bevacizumab was not toxic to corneal endothelial cells either alone or in combination with VEGF at various clinically relevant doses.
